# Acute Severe Heart Failure in Paediatric Inflammatory Multisystem Syndrome Temporally Associated With SARS-CoV-2: A Case Report

**DOI:** 10.7759/cureus.37616

**Published:** 2023-04-15

**Authors:** Muhammad Maaz Sajid, May Lynn Chan, Manal Ali, Nauman Ahmed

**Affiliations:** 1 Cardiology, Southport and Formby District General Hospital, Southport, GBR

**Keywords:** cardiogenic shock, left ventricular systolic dysfunction, asthenia, heart failure, intensive care units, covid-19 related, paediatric multisystem inflammatory disease

## Abstract

A 17-year-old boy presented during the COVID-19 pandemic in late 2021 with intractable fevers and hemodynamic instability with early gastrointestinal disturbances, resembling features of the pediatric inflammatory multisystem syndrome temporally associated with SARS-CoV-2. Our patient required intensive unit care for persistently worsening signs of cardiac failure; initial admission echocardiography demonstrated severe left ventricular dysfunction with an estimated ejection fraction of 27%. Treatment with intravenous IgG and corticosteroids showed a rapid improvement in symptoms, but further specialist cardiological input was required for heart failure in the coronary care unit. Substantial improvement in cardiac function was shown on echocardiography before discharge, initially to left ventricular ejection fraction (LVEF) 51% two days after the commencement of treatment and then to >55% four days later, and on cardiac MRI. An echocardiogram one month post-discharge was normal, and the patient reported complete resolution of heart failure symptoms by four months in addition to full restoration of functional status.

## Introduction

Clusters of children presenting with novel pediatric multi-system inflammatory conditions temporally associated with COVID-19 (hereafter PIMS-TS, per the definition offered by the Royal College of Pediatrics and Child Health) emerged during the first wave of the COVID-19 global pandemic [1]. In April 2020, in the first months of the pandemic, cases of children presenting with high fever and shock were reported in the United Kingdom [2]. Although most children infected with COVID-19 have either remained asymptomatic or suffered from a mild form of the disease, a small but clinically significant minority go on to develop severe multi-organ hyper-inflammatory involvement [3,4]. The majority of these cases required admission to intensive care units, with a sizeable proportion requiring inotropic support [5]. With the appropriate treatment, however, clinical evolution and prognosis have been generally favorable.

Acute cardiac dysfunction is now well established in children with PIMS-TS, a presentation that overlaps with other more well-known acute inflammatory syndromes such as Kawasaki disease and toxic shock syndrome [6]. Evidence of cardiac involvement has been demonstrated by biochemical, electrocardiographic, and echocardiographic data. The most commonly observed complications are shock, cardiac arrhythmias, pericardial effusion, and coronary artery dilatation in one systematic review, and cardiogenic shock was observed in the majority (up to 80%) of presentations [7,8].

In this report, we describe the case of a pediatric patient treated for PIMS-TS during the wave of the COVID-19 pandemic in late 2021, in which the greater burden of the disease process fell on severe cardiogenic shock requiring inotropic support, mainly relating to significant myocardial dysfunction concomitant with myocarditis. We provide the four-month follow-up outcome as well as a subjective account of the disease experienced by the patient.

## Case presentation

The patient was a 17-year-and-11-month-old white British boy with no significant past medical history who initially presented with his mother to the emergency department on October 8 with a persistent posterior headache and significant asthenia for four days and high temperatures for three days; he further complained of a concurrent sore throat, neck stiffness, and more recent onset bilateral otalgia. He had suffered from COVID-19 infection one month prior but had mild symptoms therefrom and recovered well.

The patient’s initial observations showed tachycardia (sinus rhythm, rate 132), tachypnea (rate 22), and pyrexia of 39.7 °C; his blood pressure and oxygen saturation readings were both normal. On examination, a further sign of blanching and an erythematous maculopapular rash extending over both wrists and both dorsal surfaces of the hands were noticed; cardiovascular and respiratory examinations were both otherwise normal. The result of the RT-PCR for SARS-CoV-2 that day was negative.

Based on the above signs and symptoms, he was diagnosed with otitis externa. He was administered intravenous paracetamol and flucloxacillin and given an oral dose of ibuprofen in the emergency department, and subsequently discharged with oral flucloxacillin, being advised to return if symptoms worsen or persist despite treatment.

He presented himself again to the department two days later with persistent symptoms and fevers, along with new diarrhea and vomiting since the aforementioned discharge. He had also since developed a mild, non-productive cough and shortness of breath, with significantly reduced functional status secondary to progressive asthenia. The maculopapular rash had now spread to the dorsal surfaces of both feet. He had no new neurological symptoms. Initial observations showed worsening sinus tachycardia (rate 139), new hypertension (187/87 mm Hg), and persistent fever (39.1 °C), but still maintaining normal oxygen saturations and a normal respiratory rate; the ECG demonstrated sinus tachycardia. Venous blood gas at this time demonstrated a lactate reading of 2.3, and there was biochemical evidence of a severe and worsening inflammatory process (Tables 1-2).

**Table 1:** Laboratory test results: biochemistry

Investigation	8-Oct	10-Oct	11-Oct	12-Oct	13-Oct	14-Oct	15-Oct	16-Oct	18-Oct
Creatinine (μmol/L)	74	78	71	60	62	63	61	64	
eGFR (ml/min/1.73 m^2^)	N/A	N/A	>90	>90	>90	>90	>90	>90	
Sodium (mmol/L)	133	134	135	138	134	139	138	139	
Potassium (mEq/L)	4.2	5.1	4.2	3.7	4.2	4.4	4.6	5.1	
Urea (mmol/L)	3.9	5	5.8	5.8	9.6	11.7	8.1	8.2	
Amylase (U/L)			38						
Vitamin B12 (pg/ml)						825			
Calcium (mmol/L)			1.86	2.06	1.98	1.96		2	
Adjusted calcium (mmol/L)			2.14	2.23	2.2	2.13		2.18	
CRP (mg/dL)	170	314	253	222	125	68		32	17
ESR (mm/hr)			17						
Ferritin (ng/mL)			1010	2860					
Folate (ng/ml)						3.3			
Free T4 (pmol/L)						15.2			
TSH (mU/L)						1.54			
LDH (U/L)					180				
ALT (IU/L)	21	66	47	163	102	79		98	
Alkaline phosphatase (IU/L)	92	102	82	75	59	58		61	
Total bilirubin (mg/dL)	13	15	11	17	10	10		11	
Albumin (g/L)	45	38	28	39	32	36		35	
Magnesium (mmol/L)			0.73	0.7	1.02	0.94		0.85	
NT-proBNP (pg/mL)			25891	31546	14508	11990		4532	
Phosphate (mmol/L)			0.81	0.99	0.71	1.42		1.16	
Procalcitonin (ng/mL)			31.39	14.22					
Total vitamin D (ng/ml)						27			
Trop T (ng/L)			157	79	58				

**Table 2:** Laboratory test results: hematology

Investigation	8-Oct	10-Oct	11-Oct	12-Oct	13-Oct	14-Oct	16-Oct	18-Oct
WCC (×1000/mm^3^)	11.1	14.8	10.7	9.7	16.6	12.8	14.3	11.4
RCC (×1,000,000/mm^3^)	4.86	4.76	3.84	3.74	3.26	3.52	4.48	4.62
Hemoglobin (g/L)	152	148	120	116	102	111	140	143
Hematocrit (%)	0.43	0.425	0.334	0.328	0.293	0.322	0.4	0.425
MCV (fL)	88.5	89.3	87	87.7	89.9	91.5	89.3	92
MCH (pg)	31.3	31.1	31.3	31	31.3	31.5	31.3	31
Platelets (×1000/mm^3^)	211	180	145	195	243	285	330	362
Neut (×10^3^ cells/μL)	9	13.4	9.4	8.4	14.8	10.6	10.2	9.3
Lymph (×10^3^ cells/μL)	0.8	0.6	0.6	1.1	1.2	1.6	3.1	1.7
Monos (×10^3^ cells/μL)	1.3	0.7	0.6	0.2	0.6	0.6	0.9	0.4
Eosos (×10^3^ cells/μL)	0.1	0.1	0	0	0	0	0	0
Prothrombin time (s)	11.7	13.5	13.8	14.5	12.2	13	13	
INR	1.1	1.3	1.3	1.4	1.2	1.3	1.3	
APTT	23		27	31	22		21	
APTT ratio	0.9		1.1	1.2	0.9		0.8	
D-dimer (ng/mL)			2598				>5000	2688
Fibrinogen (g/L)			6.8					

Early on in admission, he developed cardiogenic shock, with a heart rate persistently above 130 beats per minute and persistent profound hypotension (85/34 mmHg) despite 3.5 liters of intravenous fluid resuscitation. The admission echocardiogram demonstrated a severely depressed ejection fraction of 27%. Due to his continually worsening cardiovascular status and significantly raised inflammatory markers, he was admitted to the intensive care unit and treated for sepsis of unknown origin with a view to ruling out meningitis. While in the unit, he required vasopressor support with noradrenaline. Inotropic support with Enoximone was commenced after advice for aggressive support of cardiac function, in view of investigations demonstrating acute right ventricular failure, from regional pediatric cardiology. He additionally required supplemental oxygen of 2 L/minute via nasal cannula but independently supported his own airway throughout admission.

After extensive discussions with local and regional pediatricians, rheumatologists, and microbiologists, an autoimmune inflammatory process was suspected as the cause of the presentation, and intravenous IgG (2 g/kg), as well as methylprednisolone (10 mg/kg), were commenced. After the discussion of the case at a regional pediatric multi-disciplinary team meeting, a formal diagnosis of PIMS-TS was pronounced.

Following the administration of intravenous IgG and methylprednisolone, a rapid positive response in general status was observed; significant improvements were noted in both inflammatory indices and symptoms of asthenia; tachycardia and tachypnea had both resolved; and the patient continued to engage well with physiotherapy. Despite standard immunomodulatory treatment, however, he continued to show persistent signs and symptoms of severe heart failure, including dyspnea on minimal exertion such as self-repositioning in bed. After a total of three days in the intensive care unit, the patient was transferred to the coronary care unit in the cardiology ward for the continuation of cardiac failure management on the advice of regional pediatric specialists and local cardiologists. In the unit, heart failure pharmacotherapy with oral beta-blockers, ACE-I, and aspirin was optimized and frequent, and regular echocardiograms were performed, demonstrating rapidly recovering function relative to admission. Cardiac MRI imaging was additionally performed in the latter days of the inpatient stay, demonstrating normal wall contractility and a fully recovered ejection fraction of 72% for the LV and 77% for the RV, representing substantial recovery from the episode of acute myocarditis.

The patient was followed up in four months’ time, being asymptomatic with normal vital signs and reporting full resolution of any residual symptoms at the time of discharge. His heart failure medication was stopped six months after discharge.

Investigation

Nasopharyngeal and throat swabs performed for COVID-19 during the initial presentation at the ED on October 8 and subsequent swabs during the inpatient stay on October 10 and 11 all tested negative. The patient did test positive for SARS-CoV2 IgG. All other virology studies were likewise negative (Table 3).

**Table 3:** Laboratory test results: microbiology **Coagulase-negative Staphyloccous grown on October 09, in the absence of central intravascular or prosthetic devices, likely skin flora contaminants causing the positive result. All virology tested using Cepheid system (Xpert).

Investigation	8-Oct	10-Oct	11-Oct	12-Oct	18-Oct
Adenovirus				Negative	
Anti-streptolysin O					200
Blood cultures	**	Negative	Negative		
Carbapenemase			Negative		
C difficile			Negative		
CMV IgM				Not detected	
CMV IgG				Detected	
EB VCA IgM				Not detected	
EBV VCA IgG				Detected	
EBV NA IgG				Detected	
Faeces PCR			Negative		
Novel coronavrius	Negative	Negative	Negative		
SARS-CoV2 IgG			Detected		
Urine			WCC 23, epithelial cells 139, no growth		
Vanc VRE screen			Negative rectal swab		
Resp virus PCR (Cepheid)			Negative SARS-CoV-2 RNA	No Adenovirus DNA detected	
				No Coronavirus 229E RNA detected	
				No Coronavirus HKU1 RNA detected	
				No Coronavirus NL63 RNA detected	
				No Coronavirus OC43 RNA detected	
				No human metapneumovirus RNA detected	
				No human Rhinovirus RNA detected	
				No Influenza A RNA detected	
				No Influenza B RNA detected	
				No MERS-CoV RNA detected	
				No Parainfluenza virus 1 RNA detected	
				No Parainfluenza virus 2 RNA detected	
				No Parainfluenza virus 3 RNA detected	
				No Parainfluenza virus 4 RNA detected	
				No RSV RNA detected	
				No *B. parapertussis* DNA detected	
				No *B. pertussis* DNA detected	

The initial blood culture on October 8 was positive for coagulase-negative staphylococcus; this was most likely contaminated by skin flora, and two subsequent cultures taken on October 10 and 11 were negative.

A chest X-ray performed on October 10 demonstrated fine reticulations in the lower lung zones bilaterally; an X-ray the following day showed progressive worsening of the same. A repeat chest X-ray the following day, on October 12th, was requested due to an increased oxygen requirement and new expectoration of pink sputum. This image demonstrated cardiomegaly and bilateral effusions, as well as additional septal lines and atelectasis suggesting pulmonary edema.

An ultrasound of the abdomen on October 12 demonstrated a small amount of free fluid in the abdomen and pelvis with bilateral pleural effusions.

Cardiac troponin T on October 11 was 157 ng/L, and NT-proBNP was found to be 25,891 pg/mL. Troponin T continued to gradually drop over the course of admission after the administration of intravenous methylprednisolone and IgG. N-terminal (NT)-pro hormone BNP (NT-proBNP), however, peaked at 31,546 pg/mL the following day and gradually decreased thereafter.

On admission to the intensive therapy unit on October 11, the initial echocardiography revealed a left ventricle that was mildly dilated and had severely impaired systolic function, with a left ventricular ejection fraction (LVEF) of approximately 27%. The left atrium and right heart were within normal size limits, with right ventricular function also showing impairment. There were no signs of valvular stenosis, but at least mild mitral regurgitation and tricuspid regurgitation were demonstrated.

Two days after the commencement of treatment, the left ventricular basal inferior wall showed hypokinesis, but overall function improved, with a 51% ejection fraction. Mild mitral regurgitation persisted, and right ventricular function improved to normal. Four days after admission, echocardiography was completely normal, with no residual mitral regurgitation, a left ventricular ejection fraction >55%, and normal right ventricular size and function.

On transfer to a larger cardiac tertiary center, the patient had a cardiac MRI, which showed mild dilatation of the left ventricle with normal systolic function and an LVEF of 72%. The right ventricle was of normal size with an EF of 77%. There were no signs of significant valvular stenosis or regurgitation. Only minimal residual myocardial edema was visualized, and no myocardial fibrosis was demonstrated, representing almost full recovery from the episode of acute myocarditis.

Differential diagnosis

Otitis externa was initially suspected in the first presentation to the emergency department, and the patient was discharged with antibiotics and safety netting. On medical review during the second presentation, due to the combination of symptoms of upper airway infection, fever, neck stiffness, and headache with vomiting, the patient was treated for community-acquired pneumonia and meningitis with a view to ruling out the latter. CT head and lumbar puncture results were both negative, and meningitis was ruled out.

Due to the persistence of an intractable fever lasting for more than five days, rash, initial abdominal pain, and profound cardiovascular instability, the intensive care team discussed the case with the regional specialist pediatric center, and a presumptive diagnosis of PIMS-TS was made on October 11, with immunomodulatory therapy being initiated accordingly.

Treatment

On initial medical review, the patient initially received both intravenous ceftriaxone and piperacillin with tazobactam for suspicion of community-acquired pneumonia and meningitis. He was definitively treated with intravenous methylprednisolone and IgG, commencing October 11.

The heart failure required separate continuing treatment, including inotropic support in the ITU and then regular oral medication in the cardiology ward; this included once-daily doses of Bisoprolol, Ramipril, and Aspirin as outlined below, in addition to a reduced dose of oral Prednisolone. Further intravenous diuretic therapy with furosemide is necessary on ITU to treat persistent pulmonary edema. The patient maintained his own airway throughout admission but had an oxygen requirement of 2 L/minute via nasal cannula in ITU.

Outcome and follow-up

The patient spent a total of three days in the ITU and was thereafter discharged to the coronary care unit for optimization of heart failure. Following seven days in the cardiology ward, he was transferred to the tertiary cardiac center for a cardiac MRI; outpatient clinic follow-up was arranged; and he was discharged with Bisoprolol 1.25 mg/day and Ramipril 2.5 mg/day to continue until otherwise advised and a course of 75 mg/day aspirin for six weeks. Tapering doses of oral steroids were provided before discharge. The advice was to restrict strenuous physical activity, including sports, for at least six months after discharge.

He was followed up in the clinic in four months’ time and was completely asymptomatic. His repeat outpatient echocardiogram was normal, with no residual valvular or wall motion abnormalities. The aforementioned heart failure medication was stopped six months post-discharge, and the patient remained very well, having recovered his functional status to the pre-admission baseline.

## Discussion

Cardiac involvement is the most common finding in PIMS-TS and comprises a significant burden of disease [8]. An early case series and analysis of 58 children in the UK identified cardiovascular collapse as one of three main clinical patterns, with a majority of those who developed shock also demonstrating evidence of acute heart failure on echocardiography [9]. A more recent Italian retrospective study of 32 children reported cardiac involvement in 26 of them (81%), of which 16% exhibited severe functional impairment on echocardiography [10]. This is consistent with almost every published retrospective cohort study, where prominent cardiac involvement is a main feature of the disease and poses a challenge to management [8].

The initial presentation in our case was unusual in a few respects. First, in addition to the mucocutaneous, neuropsychiatric, and hemodynamic manifestations described, gastrointestinal involvement presented later on and constituted a minor portion of the overall clinical burden. In retrospect, cardiac involvement was likely a part of the first presentation, accounting for the patient’s tachycardia and worsening asthenia, which had significantly deteriorated by the second emergency department presentation.

Furthermore, three consecutive daily SARS-CoV-2 RT-PCR tests and a feces PCR test all resulted in negatives with concurrently positive SARS-CoV-2 IgG serology. This has been noted in previous case studies [11], suggesting that this clinical presentation may have been a post-infectious phenomenon. We note, however, that in the large case series by Feldstein et al., most patients with severe cardiovascular involvement were antibody-positive but not necessarily RT-PCR negative [12]. In the context of this, and given the first emergency presentation of otalgia and pharyngitis symptoms, it may be that PIMS-TS developed in a later stage of COVID-19 illness, where the detectable viral load was subclinical.

The case we present here stands out chiefly because of the severity of cardiac involvement in an otherwise fit and healthy adolescent male. The admission echocardiogram demonstrated severely impaired left systolic dysfunction with an ejection fraction of approximately 27%; this degree of depression has been identified in the minority of patients with PIMS-TS myocardial dysfunction [5]. Additionally, we observed a severely elevated NT-proBNP of 31546 pg/ml, which is more than double the value of mean NT-proBNP levels in similar patients as identified in the systematic review of Henrina et al. involving 1228 pooled subjects [13]. Troponin T was 157 ng/L, which is also considerably higher than the mean value of 75 [13]. This biochemical and echocardiographic picture was consistent with the aforementioned X-ray findings, worsening edematous signs, and rapidly declining patient general status prior to the commencement of IV IgG and steroid therapy. Interestingly, our patient only demonstrated sinus tachycardia during the earlier days of his inpatient stay with no other ECG abnormalities; this contrasts with the majority of similar cases in which abnormalities such as low QRS amplitudes and transient T-wave inversions were commonly observed [14]. Additionally, no aneurysm formation was identified.

Mannarino et al. noted that derangements in the inflammatory indices, namely CRP, white blood cells, and neutrophils, were significantly higher in patients with more severe cardiac involvement on echocardiography [10]. Troponin T and NT-proBNP levels were likewise higher in patients with severely depressed function. In patients with shock but normal or mildly depressed ejection fraction, high NT-proBNP levels may indicate a hyperinflammatory state, as inflammatory states have an association with natriuretic peptide release [15]. In our patient, however, it was likely a combination of hyperinflammation and severe acute heart failure as independent factors due to persistent signs and symptoms even after the administration of immunoglobulins and methylprednisolone. Further specialized monitoring and treatment were required before biochemical and clinical evidence of improvement became apparent.

Our patient was an older adolescent male who had turned 18 years of age during his inpatient stay, which is consistent with the greater recorded involvement of older children and adolescents in PIMS-TS [16], as well as the predominance of the male sex [13]. Also in accordance with previous studies, a prompt positive response was observed to intravenous steroid and immunoglobulin therapy [17,18]. His LVEF was found to have normalized before discharge; this is in line with prior studies showing that the likelihood and trajectory of LVEF normalization in patients with severe LVEF depression have been similar to those with mild dysfunction, averaging one to two weeks [5,19]. Six months post-discharge, the patient reported full recovery with no residual symptoms.

## Conclusions

Though PIMS-TS remains a rare COVID-19-associated syndrome, it can cause life-threatening cardiovascular complications and have potential long-term sequelae if a timely diagnosis is not made. Though our patient was promptly diagnosed and treated, he required further treatment for heart failure with cardiac monitoring, both in the ITU with inotropic support and in the coronary care unit. Although the consequent heart failure in PIMS-TS is uncommonly severe, cases such as the one we present here have been noted, albeit rarely, in previous literature. Due clinical diligence is required in identifying the degree of cardiovascular involvement, including evaluations with echocardiography, ECG, and laboratory cardiac indices, and treating accordingly to increase the likelihood of a favorable outcome.

If older adolescents with a recent history of COVID-19 but no other underlying disease experience new cardiac symptoms of heart failure that do not respond to initial therapy, PIMS-TS should be suspected. Early multidisciplinary involvement can facilitate a more rapid diagnosis and improve the prognosis. Despite its critical nature, the prognosis is excellent if PIMS-TS is identified early and treated promptly with immunoglobulins and corticosteroids. Long-term follow-up is strongly recommended to monitor the restoration of cardiac function and possible sequelae.
